# Assessment of Bacterial Diversity of Industrial Poultry Wastewater by Denaturing Gradient Gel Electrophoresis (DGGE) and the Cultivation Method in Order to Inform Its Reuse in Agriculture

**DOI:** 10.1155/2022/6065305

**Published:** 2022-09-20

**Authors:** Amira Oueslati, Wafa Hassen, Ali Ellafi, Sana Alibi, Ahlem Jaziri, Sarra Bachkouel, Imen Oueslati, Mejdi Snoussi, Mohd Adnan, Mitesh Patel, Abdelbaset Mohamed Elasbali, Hedi Ben Mansour

**Affiliations:** ^1^Research Unit of Analysis and Process Applied on the Environmental-APAE UR17ES32-Higher Institute of Applied Sciences and Technology Mahdia, University of Monastir, Tunisia; ^2^Higher Institute of Biotechnology of Monastir, University of Monastir, Tunisia; ^3^Laboratory of Analysis, Treatment and Valorization of the Pollutants of the Environment and Products, Faculty of Pharmacy, University of Monastir, Avicenne Street, Monastir 5000, Tunisia; ^4^Faculty of Sciences of Gafsa, Campus Universitaire Sidi Ahmed Zarroug, University of Gafsa, Gafsa 2112, Tunisia; ^5^Specialized Unit Support for Research and Technological Transfer US19CBBC01, Borj-Cédria Biotechnology Center, B.P. 901, 2050 Hammam-Lif, Tunisia; ^6^Department of Biology, College of Science, University of Hail, Hail, P.O. Box 2440, Saudi Arabia; ^7^Laboratory of Genetics, Biodiversity and Valorization of Bio-Resources (LR11ES41), Higher Institute of Biotechnology of Monastir, University of Monastir, Avenue Tahar Haddad, BP74, Monastir 5000, Tunisia; ^8^Department of Biotechnology, Parul Institute of Applied Sciences and Centre of Research for Development, Parul University, Vadodara, India; ^9^Department of Clinical Laboratory Science, College of Applied Sciences-Qurayyat, Jouf University, Saudi Arabia

## Abstract

Effluents discharged by poultry meat industries are heavily polluted with raw materials, such as fat, blood residues, and proteins. Thus, untreated effluents directly discharged into the environment may constitute a public health threat. This study aims to evaluate the bacterial diversity of three water qualities: industrial poultry wastewater (PWW), tap water (TW), and PWW diluted with TW (50 : 50) (V/V) (TWPWW) by the combination of culture-independent and culture-dependent approaches. The total bacterial DNA was extracted using phenol/chloroform method. The hypervariable 16S rRNA region V3-V5 was amplified by PCR using universal primers. The amplicons were separated by vertical electrophoresis on a polyacrylamide gel of increasing denaturing gradient according to their richness in GC bases. Selected bands were reamplified and sequenced. Pure isolated bacteria from nutrient agar medium were characterized according to their morphological and biochemical characteristics. Genomic DNA from pure strains was extracted by boiling method, and a molecular amplification of the 16S–23S ITS region of the 16S rRNA gene was performed using the universal primers. Selected isolates were identified by sequencing. Results showed a high bacterial load and diversity in PWW in comparison with TW and TWPWW. A collection of 44 strains was obtained, and 25 of them were identified by sequencing. *Proteobacteria* represented 76% of isolated bacteria *Gamma-Proteobacteria* was the predominate isolate (68%). Other isolates were *Firmicutes* (8%), *Bacteroidetes* (12%), and *Actinobacteria* (8%). These isolates belong to different genera, namely, *Pseudomonas*, *Acinetobacter*, *Proteus*, *Empedobacter*, *Corynebacterium*, *Enterobacter*, *Comamonas*, *Frondibacter*, *Leclercia*, *Staphylococcus*, *Atlantibacter*, *Klebsiella*, and *Microbacterium*.

## 1. Introduction

The increase in population means an increase for food demand. Currently, the poultry meat and egg products industries are considered as one of the most important and fastest growing agri-food industries [1–3]. Generally, industrial process activities are associated with the use of large amount of freshwater given that all production operations require hygiene and quality control [4–6]. It has been estimated that the water consumption average is around 26.5 L per bird, which remains dependent on the degree of automation [7, 8]. As a consequence, large quantities of highly polluted wastewater are generated [2, 9]. These effluents were classified among the most polluted discharges, due to the high concentration of physico-chemical properties including chemical oxygen demand (COD), biochemical oxygen demand (BOD), and total suspended solids (TSS), as well as nutritive elements (nitrogen and phosphorus) and organic matter including proteins from blood residues and fats from slaughtering and cleaning activities [9–13]. Besides their organic and inorganic load, poultry slaughter houses have shown the presence of a high load of pathogenic (*Pseudomonas aeruginosa*, *Shigella*, *Salmonella*, *Escherichia coli*, *Vibrio cholerae*, and *Brucella*) [14–16] and nonpathogenic bacteria such as total and fecal coliforms of which *Aeromonas* spp. and *Clostridium* spp. are the two main indicators, as well as strains belonging to the group of *Streptococcus* [17, 18]. It has been reported that in developing countries, abattoirs are generally located near rivers [19, 20], and untreated effluents directly released into the environment without any treatment or after primary treatment only [21], allowing to reduce the effluent load of fats and suspended solids [17], but not the microbiological risk [22, 23]. This direct discharge increases the contamination level by pathogenic bacteria, leading to a serious environmental problems and human pathogens' transmission [24].

Currently, in order to investigate the microbial diversity in a given ecosystem, culture-dependent or culture-independent approaches can be applied [25]. However, the use of molecular techniques for microbial community characterization is recommended over the traditional methods, which allows only the identification of cultivable microorganisms. In fact, the use of artificial homogenous medium disadvantage is allowing the growth of only a small fraction of cultivable microorganisms. Moreover, enumerating bacterial results may be inaccurate, since the bacteria can only be cultivated under optimal growth conditions [25–27]. In contrast, especially uncultivable bacteria can be detected by molecular techniques, such as 16S rRNA-based methods, as well as those present in low abundance or growing so slowly that traditional culture-based protocols cannot determine them [28].

Recent studies recommended the use of molecular techniques based on fingerprinting characteristics to target the diversity of the universal gene 16S rRNA, and they seem to be ideal for community comparison [29, 30, 31]. Among these techniques, denaturing gradient gel electrophoresis (DGGE) has been previously adopted by many scientists for microbial analysis of wastewater and poultry abattoir effluents. It was considered as a potential fingerprinting technique of microbial community composition, diversity, and dynamics [29, 32]. This work was aimed to study and assess the bacterial diversity of industrial poultry wastewater by the combination of culture-independent and culture-dependent techniques. This work was carried out within the framework of a valorization of industrial wastewater in the irrigation of olive trees. Indeed, previous studies have shown that the reuse of industrial wastewater from the food industry contributes to the stimulation of the vegetative growth of young olive trees and to the germination of wheat seeds [33, 34]. The microbial characterization of this industrial wastewater will be compared with the water used as control.

## 2. Material and Methods

### 2.1. Sampling and Preparation

Samples were collected from a poultry slaughter house located in the Government of Mahdia, in the Middle East of Tunisia (N35° 28′ 11^″^, E10° 57′ 23^″^). The samples of wastewater (PWW; collected in the morning, when the slaughtering was performed) and tap water (TW) were, respectively, collected in a sterile glass bottles. Wastewater sample was diluted with tap water V/V (50 : 50) (TWPWW). It should be mentioned that the industrial wastewater collected is not treated, but a blood separation was carried out before discharging. Samples were transferred to the laboratory immediately and stored at +4°C.

### 2.2. Culture-Independent Approach

#### 2.2.1. Extraction of Total DNA and PCR Amplification

For each sample, 800 ml was filtered immediately after sampling in sterile conditions through a sterile cellulose nitrate membrane using different pore sizes (0.8, 0.45, and 0.22 *μ*m). The aim of using different pore sizes filters was to sequester different sizes of bacteria. Total DNA was extracted as described with slight modifications [35]. Ethanol was used to wash the extracted DNA. Then, the DNA was dissolved in Tris EDTA buffer. The molecular size and the concentration of DNA were determined by agarose gel electrophoresis.

The amplification of a hypervariable 16S rRNA V3-V5 region was performed in a final volume reaction of 30 *μ*l containing 15 *μ*l of commercialized mix (Gene On, Ludwigshafen am Rhein, Germany), 0.24 *μ*l of forward primer F-357-GC5′-TACGGGAGGCAGCAG-3′, 0.24 *μ*l of reverse primer R-9075′-CCGTCAATTCCTTTGAGTTT-3′ [36], and 1 *μ*l of appropriately diluted template DNA. The initial denaturing step was performed at 94°C for 3 min, followed by 10 cycles of 94°C for 30 s, 61°C for 1 min, and 72°C for 1 min, 20 cycles of 94°C for 30 s, 56°C for 1 min, and 72°C for 1 min, and a final extension at 72°C for 10 min. Amplicon (620 bp) was migrated in 2% agarose gel in 0.5× TBE buffer and visualized under UV light.

#### 2.2.2. Denaturing Gradient Gel Electrophoresis (DGGE) Analysis

DGGE analysis was conducted with kuroGEL 2020 (VWR International bvba, USA). Amplified DNA was posed on 7% polyacrylamide gel in Tris-acetate-EDTA buffer (TAE 1×). The denaturing gradient (formamide/urea) ranged from 40 to 60%. Gels were run in constant conditions of temperature (60°C) and voltage (99 V) for 20 hours. After electrophoresis, visualizing was performed by staining the gel in ethidium bromide solution for 15 min and then washed with sterile distilled water, and gel photos were photographed under UV. Obtained DNA bands were cut and eluted in 80 *μ*l of sterile distilled water and preserved at -20°C for further utilization. Before sequencing, the eluted DNA fragments were again reamplified using unclamped 907R and 357F primers [31]. Obtained sequences were submitted in the GenBank. DGGE profiles were exploited to create matrices indicating the presence or absence of bands, and a dendrogram was built by multivariate statistical package software (MVSP), which uses the UPGMA algorithm (unweighted pair group method with arithmetic mean) and the Jaccard's coefficient.

#### 2.2.3. Culture-Dependent Approach

*(1) Bacterial Isolation*. In order to isolate a pure bacterial strain, 1 ml of each sample (TW, PWW, and TWPWW) was diluted from 10^−1^ to 10^−8^ in sterile NaCl 0.9% (w/v) and spread out in duplicate for greater accuracy in solid nutrient agar. Petri dishes were subsequently incubated for 48 h at 30°C. The number of colonies were counted and expressed as colony-forming unit (CFU) per ml. Purified individual colonies were selected according to their morphological characteristics. For all the isolates, Gram staining and catalase and oxidase tests were performed and were finally stored in 25% glycerol solution at -20°C.

*(2) Taxonomical Identification of Bacterial Isolates*. Genomic DNA from pure strains was extracted by boiling method with minor modifications [37]. Briefly, the bacterial pellets were suspended in 200 *μ*l of TE buffer (Tris-HCl [10 mM]: EDTA [1 mM]), followed by vigorous homogenization by vortexing for 30 s. The suspensions were subjected at 100°C in a boiling water-bath for 10 min. Immediately after boiling, the microfuge tubes were placed in an ice-bath for 5 minutes. After centrifugation, the supernatant containing DNA was transferred to another clean tube and stored at –20°C until analysis. Molecular amplification of the 16S–23S ITS region and the 16S rRNA gene was performed as described [38, 39], using the universal primers S-D-Bact-1494-a-20 (GTCGTAACAAGGTAGCCGTA), L-D-Bact-0035-a-15 (CAAGGCATCCACCGT), S-D-Bact-0008-a-S-20 (CTACGGCTACCTTGTTACGA), and S-D-Bact-1495-a-S-20 (AGATTTGATCCTGGCTCAG). All the PCR products (ITS and 16S rRNA amplicons) were migrated, respectively, on standard 2% agarose gels in 0.5× Tris-borate-EDTA buffer and stained for 20 min in ethidium bromide solution (0.5 mg/l). Amplification of 16S rRNA fragments was followed by sequencing, and then, obtained sequences were aligned and identified by comparing with those available at the National Centre for Biotechnology Information (NCBI) database (http://www.ncbi.nlm.nih.gov) using the BLAST program [40]. Neighbor joining method was used to construct a phylogenetic dendrogram, and tree topology was evaluated by performing boot-strap analysis of 1,000 data sets using MEGA 6 software [41]. The sequences reported in this study have been submitted to NCBI GenBank, and the accession numbers are listed in Table 1.

## 3. Results and Discussion

### 3.1. Bacterial Community Structure of the Poultry Wastewater

**Figure 1:**
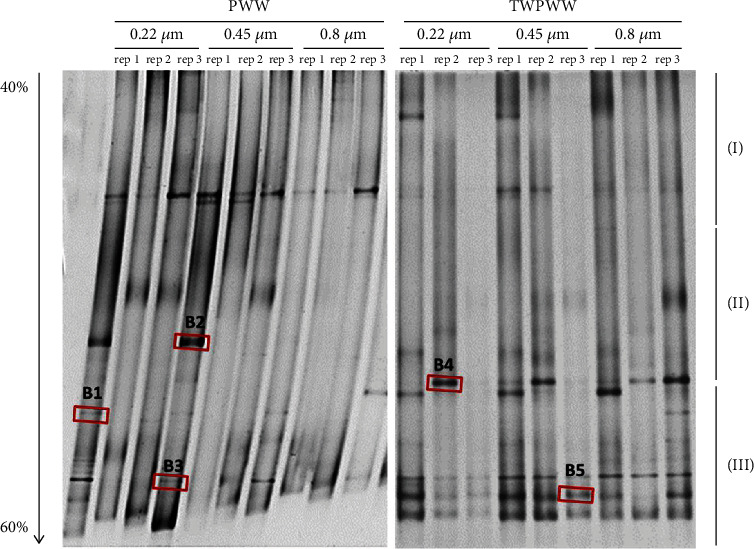
DGGE profiles of PCR products obtained from PWW and TWPWW samples showing the variation of the bacterial population based on variable region V3–V5 of 16S rDNA. Three types of bands were defined, with correlation to the running level, short (I), medium (II), and long (III) migration bands. Marked bands were excised and sequenced. The urea and formamide gradient ranged from 40 to 60%.

The V3-V5 hypervariable region analyzed by DGGE method gave a general idea of the bacterial community of PWW and TWPWW samples, and the DGGE analysis targeting the V3–V5 hypervariable region of the 16S rRNA was performed. The different sample profiles obtained after filtration through different filters diameters are shown in Figure 1. In this study, we detected many bands with different migration distances and intensities. Based on the visual analysis, DGGE profiles can be divided into three sections: short migration (I) strains with nonrich GC bonds, medium migration (II) strains moderately rich in GC bonds, and long migration (III) strains rich in GC bonds. Some bands were common in all samples, especially in the third section of TWPWW. In fact, fragments of 16S rRNA, obtained by long migration, seemed to be predominant (Figure 1).

**Figure 2:**
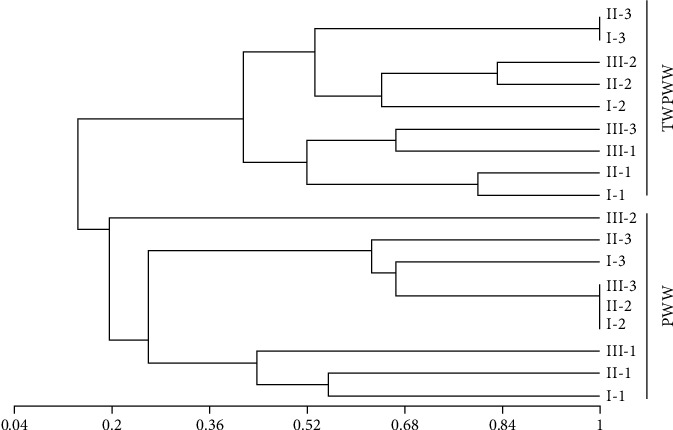
Cluster analysis showing the degree of similarity (Jaccard's coefficient) of bacterial DGGE profiles of PWW and TWPWW samples (I =0.22 *μ*m, II =0.45 *μ*m, III =0.8 *μ*m); 1-3: number of repetitions.

**Figure 3:**
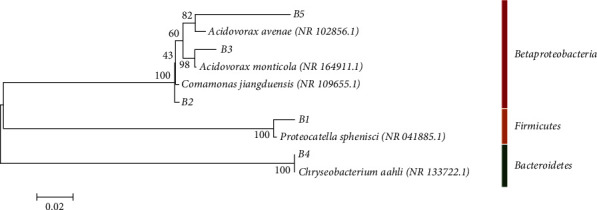
Phylogenetic trees of bacterial 16S rRNA sequences retrieved from the wastewater samples. Phylogenetic dendrogram was evaluated by performing bootstrap analysis of 1,000 data sets using MEGA 6.

In order to estimate the DGGE profile similarity between PWW and TWPWW, a cluster analysis was performed. Results showed two definite clusters with 0.197 of similarity according to Jaccard's coefficient. The two profiles obtained after filtration of the samples through 0.22- and 0.45-*μ*m filters presented the greatest similarities. These results could be in part due to the sequestration of bacteria during filtration through the 0.45-*μ*m diameter filter, due to clogging of the filter by the colloidal material (Figure 2). Five bands were excised from the gel and were sequenced and analyzed (Figure 1, Table 1). The selected bands were common in different samples. The five DGGE bands were identified as *Proteocatella sphenisci* (B1), *Comamonas jiangduensis* (B2), *Acidovorax monticola* (B3), *Chryseobacterium aahli* (B4), and *Acidovorax monticola* (B5) (Figure 3). B1 was excised from PWW sample, and it was common in all different filter diameter profiles with a low intensity. Results indicated that it was affiliated to *Proteocatella sphenisci*, which belongs to *Peptostreptococcaceae* family characterized by anaerobes and fermentative metabolism [42]. Few bibliographic databases are available on *P. sphenisci*; however, it has been isolated from a sample of guano of the Magellanic penguin (*Spheniscus magellanicus*) in Chilean Patagonia. The study mentioned the tolerance of this strain to low temperature degrees (down to +2°C) [43]. This tolerance may be the origin of its persistence even after meat cooling, which may explain its presence in PWW effluent. The same study described different profiles of resistance to antibiotics of *P. sphenisci*, and the results showed a high resistance to ampicillin (250 *μ*g/ml) versus a sensitivity to tetracycline, kanamycin, rifampicin, gentamicin, vancomycin (250 *μ*g/ml), and chloramphenicol (125 *μ*g/ml). In a previous work, two strains (*Peptostreptococcus russellii* and *Peptostreptococcus anaerobius*) belonging to *Peptostreptococcaceae* family were identified in red meat abattoir wastewater by DGGE approach [32].

B2 was detected in PWW sample, and it was a common band in 0.22 and 0.45 *μ*m profiles, and the results indicated that was affiliated to *Comamonas jiangduensis*. The genus belongs to *Comamonadaceae* family. The species was isolated for the first time from agricultural soil [44]. B3 and B5 were a common band in PWW and TWPWW samples. The BLAST results affiliated the nucleotide sequence to *Acidovorax monticola.* The strain belongs also to *Comamonadaceae* family, and it has been considered as biotrophic pathogen [45, 46]. The *Comamonas* genus has been described as one of the most abundant members of microbial communities in different natural environments [47–49]. In South Africa, two previous studies mentioned the presence of *Comamonas* sp. and *C. denitrificans* in poultry slaughter house effluents by applying, respectively, classic isolation and the fingerprinting technique DGGE [9, 32]. Few bibliographic data have evaluated the pattern of antibiotic resistance in *Comamonas* species. *C. jiangduensis* was found to be highly resistant to erythromycin with a minimum inhibitory concentration of 512 *μ*g/ml [50]. In general cases, bacteria belonged to the genus *Comamonas* which is a nonpathogenic bacterium, rarely opportunistic. However, some species were reported as responsible of severe diseases such as bacteremia, appendicitis, and meningitis [51–53].

B4 was common in all TWPWW profiles, and the sequence was affiliated to *Chryseobacterium aahli* with 99.82% of similarity. The genus *Chryseobacterium* belongs to *Flavobacteriaceae* family, and it was isolated from various natural environments [54–56] including plants, soil, water, sludge, and human [57–59] and a common colonizer of some foods, like milk, fish, meat, and poultry [57, 60]. It has been reported that the genus *Chryseobacterium* was generally associated to food deterioration [61, 62], which implies extracellular enzymes like proteases and lipases [63], and this may explain its occurrence in food environment. It has reported that poultry feathers have been shown as a shelter for *Chryseobacterium* strains with very high keratinolytic activity [64]. Previous studies showed the presence of *Chryseobacterium* genus in raw chicken [60, 65] and apparently in living and healthy chicken [66]. The strains of *Flavobacteriaceae* family can be associated to many infections especially in birds [67] and humans [68].

### 3.2. Isolation and Identification of Bacterial Isolates

Water samples were enumerated by cultivating the isolates on nutrient agar medium. Results showed that the number of cultivable bacteria was higher in PWW sample (1.4 10^5^ ± 1.8 10^4^) and lower in TW sample (2.6 10^4^ ± 1.1 10^3^). TWPWW sample presented an intermediate value (Table 2). The microbiological richness of the industrial wastewater compared to the other two samples TWPWW and TW may be attributed to the high concentration of physico-chemical parameters (TSS, COD, DOB, TOC, and NO_3_^−^) of this effluent, which is already carried out in a previous study [69]. The high level of BOD is a marker of the biological oxidation of organic compounds due to the high bacterial load [70]. In fact, the wastewater generated from the slaughter houses was classified as heavily polluted wastes, due to their high physico-chemical parameters concentration as well as nutrients (nitrogen and phosphorus) and organic matter including proteins from blood residues and fats [70–72]. According to one study, the organic matter plays the role of a growth medium for bacterial multiplication [73]. Besides, a positive correlation was established between total nitrogen and total phosphorus concentration and the microbial load [74]. In addition, it has been described that TSS serve as adsorption surface for microorganism [75–79] by establishing van der Waals and electrostatic forces [80]. The results obtained are in agreement with those already found in a previous study, where the use of PWW in the irrigation of young olive trees showed a decrease in the organic matter content in the soil in comparison with the soil irrigated with TW. These results have been attributed to the increased biological activity [81].

**Figure 4:**
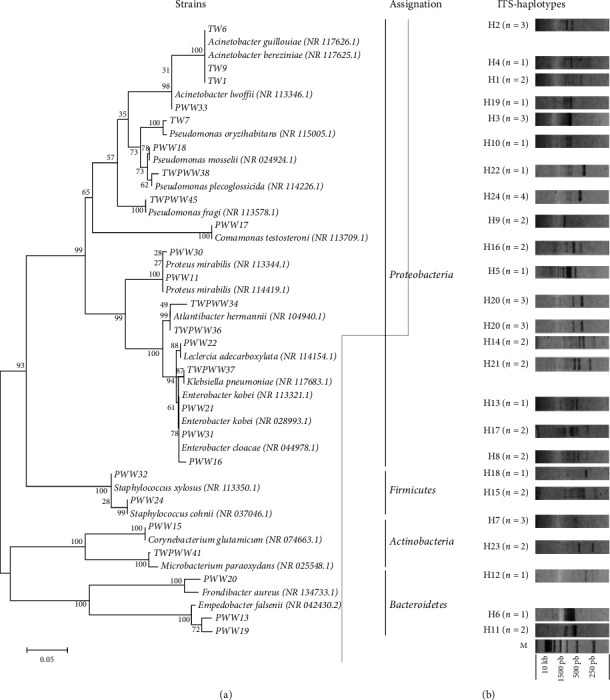
Phylogenetic diversity of bacterial isolates based on 16S rRNA partial sequences. (a) Phylogenetic dendrogram of 25 partial 16S rRNA sequences was evaluated by performing bootstrap analysis of 1,000 data sets using MEGA 6. Accession numbers of the reference strains 16S rRNA sequences are in parenthesis. (b) 16S–23S rRNA ITS haplotypes of 25 representative isolates as resolved on 2% agarose gels. ITS haplotype numbers and the number of isolates per ITS haplotype are indicated. M: molecular size marker 1Kb.

A collection of 44 strains was obtained. The selection of pure strains was based on their morphological characteristics and catalase and oxidase activities, as well as the Gram reaction (Table 3). ITS-PCR fingerprinting was used to elucidate the diversity of bacterial collection. In this work, 24 different haplotypes were obtained indicating an important bacterial diversity and including four strains recovered from TW, fifteen strains from PWW, and five strains from TWPWW samples (Figure 4(a)). The ITS-PCR profiles contained 1–5 reproducible bands with sizes ranging from 250 to about 1000 bp. Sequencing of partial 16S rRNA gene was executed for representative bacterial isolates of each distinct haplotype (*n* = 24) and was analyzed by BLAST algorithm (Table 3). The majority of bacterial isolates (76%) belonged to *Proteobacteria* (with a predominance of *Gamma-Proteobacteria*, 68%), while the remaining isolates were affiliated with *Firmicutes* (8%), *Bacteroidetes* (12%), and *Actinobacteria* (8%). These isolates were affiliated to 13 different genera including *Pseudomonas*, *Acinetobacter*, *Proteus*, *Empedobacter*, *Corynebacterium*, *Enterobacter*, *Comamonas*, *Frondibacter*, *Leclercia*, *Staphylococcus*, *Atlantibacter*, *Klebsiella*, and *Microbacterium.*

Based on the phylogenetic analysis (Figure 4(b)), the strains TW1, TW6, and TW9 were affiliated to the genus *Acinetobacter*. *Acinetobacter* species are ubiquitous, and they occupy diverse environments such as soils, fresh water, oceans, sediments, and contaminated sites [82–86]. In the past, this genus was considered to be an organism of low virulence [87]; however, it has recently attracted the attention of scientists and clinicians, in terms of their fundamental biological properties and pathogenic potential [88]. Previous studies showed the presence of four isolates in surface waters belonging to *Acinetobacter genus* with multiresistance to antibiotics [89]. The presence of *Pseudomonas oryzihabitans* was previously mentioned in the environment; however, its presence on suspended particulate water matters was described for the first time in 2000 with a high resistance to chlorine [90]. This bacterium does not belong to the normal human flora. Nowadays, this bacterium is considered as a pathogenic human bacterium, and several studies indicated that bacterium's transition is through environment [91–95].

**Figure 5:**
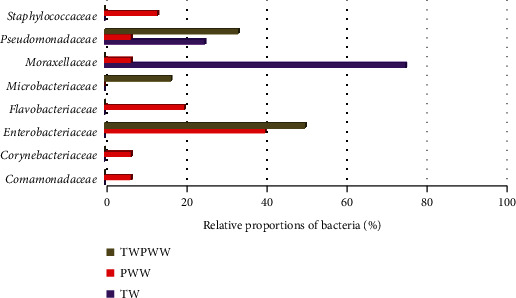
Occurrence of the different families of bacteria isolated from different water samples. TW: tap water; PWW: poultry wastewater; TWPWW: diluted poultry wastewater sample with tap water V/V (50 : 50).

Wastewater generated by slaughter houses is potentially contaminated with bacteria resistant to antibiotics [17]. In this study, the occurrence of the different isolate families from PWW samples showed the dominance of *Enterobacteriaceae* (40%) followed by *Flavobacteriaceae* (20%) and *Staphylococcaceae* (13.3%) (Figure 5). According to the bibliography, genus belonging to *Enterobacteriaceae* family has been detected either in poultry meat or in poultry slaughter house wastewater. Those results are expected since most of them are part of the intestinal microbial flora of healthy animals [96]. German studies have recently demonstrated the occurrence of colistin resistant *Enterobacteriaceae* (*E. cloacae* complex, *E. coli*, and *K. pneumoniae*) in process waters and wastewater from poultry slaughter houses [96, 97]. Several authors reported the presence of *Pseudomonas mirabilis* in poultry meat and chicken droppings [98–100]. *P. mirabilis* is known as an opportunistic pathogen that causes human urinary tract and nosocomial and wound infections [101]. Skin chilled poultry was a reservoir for *Klebsiella oxytoca*, *Klebsiella* sp., *Leclercia adecarboxylata*, and *Pseudomonadaceae* (*P. fragi* and *P. putida*) [102]. In Selangor, *Staphylococcus aureus* was isolated from poultry slaughter house wastewater with high antimicrobial resistance [103].

In the light of the results found, the dependent culture technique made it possible to isolate a greater number of bacteria than the independent culture technique which belongs to several families. However, the adoption of the DGGE technique revealed that the sequenced strains belong to only three families, of which the *P. sphenisci* strain was the only strain detected among the two techniques. In addition, this technique showed an abundance of strains belonging to *Comamonadaceae* contrary to culture-dependent technique where *Enterobacteriaceae* was the dominant family. In general context, recent microbial molecular approaches can be adopted in order to have an exceptional information about microbial communities [104].

## 4. Conclusion

This research demonstrated that the combination of two approaches, culture-dependent and culture-independent techniques, provides a more precise idea of the microbial community and diversity. The findings showed that the situation is alarming, since pathogenic bacteria may contaminate downstream water source, which can be the cause of environment and food contamination. The governmental authorities are invited to better control the quality of these discharges before their evacuation in the receiving environment by the establishment of sophisticated treatment processes which allow the elimination of pathogenic bacterial strains.

## Figures and Tables

**Table 1:** 16S rRNA V3-V5 sequence similarities of the excised bands to the closest relatives retrieved from GenBank.

DGGE bands	Sample	Filter diameter (*μ*m)	Accession number	Closest species	Phylogenetic affiliation	Homology (%)	Length (bp)
B1	PWW	0.22	OL636138	*Proteocatella sphenisci*	*Peptostreptococcaceae*	99	494
B2	PWW	0.45	OL636139	*Comamonas jiangduensis*	*Comamonadaceae*	99.26	557
B3	PWW	0.45	OL636140	*Acidovorax monticola*	*Comamonadaceae*	99	580
B4	TWPWW	0.22	OL636141	*Chryseobacterium aahli*	*Flavobacteriaceae*	99.82	551
B5	TWPWW	0.45	OL636142	*Acidovorax avenae*	*Comamonadaceae*	97	430

**Table 2:** Enumeration of total biomass.

Sample	C_N_ (CFU/ml)	Standard deviation (±SD)
*TW*	2.6 10^4^	1.1 10^3^
*PWW*	1.4 10^5^	1.8 10^4^
*TWPWW*	4.910^4^	1.7 10^3^

**Table 3:** Identification and biochemical characteristics of bacterial strains isolated from different water samples.

Isolates	Accession number	Closest relative	Sequence similarity (%)	Length (bp)	Phylogenetic affiliation	Gram strain	Catalase	Oxidase
TW1	OL636143	*Acinetobacter bereziniae*	99.72	727	*Moraxellaceae*	G-	+	—
TW6	OL636144	*Acinetobacter guillouiae*	99.63	812	*Moraxellaceae*	G-	+	—
TW7	OL636145	*Pseudomonas oryzihabitans*	99	704	*Pseudomonadaceae*	G-	+	—
TW9	OL636146	*Acinetobacter bereziniae*	99.42	686	*Moraxellaceae*	G-	+	—
PWW11	OL636147	*Proteus mirabilis*	99.72	710	*Enterobacteriaceae*	G-	+	—
PWW13	OL636148	*Empedobacter falsenii*	99	689	*Flavobacteriaceae*	G-	+	+
PWW15	OL636149	*Corynebacterium glutamicum*	99	674	*Corynebacteriaceae*	G+	+	—
PWW16	OL636150	*Enterobacter cloacae*	100	838	*Enterobacteriaceae*	G-	+	—
PWW17	OL636151	*Comamonas testosteroni*	99.42	855	*Comamonadaceae*	G-	+	+
PWW18	OL636152	*Pseudomonas mosselii*	99.85	686	*Pseudomonadaceae*	G-	+	+
PWW19	OL636153	*Empedobacter falsenii*	98	710	*Flavobacteriaceae*	G-	+	+
PWW20	OL636154	*Frondibacter aureus*	95.39	328	*Flavobacteriaceae*	G-	+	+
PWW21	OL636155	*Enterobacter kobei*	99.48	388	*Enterobacteriaceae*	G-	+	—
PWW22	OL636156	*Leclercia adecarboxylata*	99.56	687	*Enterobacteriaceae*	G-	+	—
PWW24	OL636157	*Staphylococcus cohnii*	99.69	637	*Staphylococcaceae*	G+	+	—
PWW30	OL636158	*Proteus mirabilis*	99.40	672	*Enterobacteriaceae*	G-	+	—
PWW31	OL636159	*Enterobacter kobei*	99.43	702	*Enterobacteriaceae*	G-	+	—
PWW32	OL636160	*Staphylococcus xylosus*	99.43	699	*Staphylococcaceae*	G+	+	—
PWW33	OL636161	*Acinetobacter lwoffii*	99.55	662	*Moraxellaceae*	G-	+	—
TWPWW34	OL636162	*Atlantibacter hermannii*	99	676	*Enterobacteriaceae*	G-	+	—
TWPWW36	OL636163	*Atlantibacter hermannii*	99.47	560	*Enterobacteriaceae*	G-	+	—
TWPWW37	OL636164	*Klebsiella pneumoniae*	100	665	*Enterobacteriaceae*	G-	+	—
TWPWW38	OL636165	*Pseudomonas plecoglossicida*	99.30	711	*Pseudomonadaceae*	G-	+	+
TWPWW41	OL636166	*Microbacterium paraoxydans*	99	678	*Microbacteriaceae*	G+	+	—
TWPWW45	OL636167	*Pseudomonas fragi*	99.26	680	*Pseudomonadaceae*	G-	+	+

G: gram; (+): positive activity; (-) negative activity.

## Data Availability

All data are presented in this manuscript.
